# Microfluidic setup for on-line SERS monitoring using laser induced nanoparticle spots as SERS active substrate

**DOI:** 10.3762/bjnano.8.26

**Published:** 2017-01-24

**Authors:** Oana-M Buja, Ovidiu D Gordan, Nicolae Leopold, Andreas Morschhauser, Jörg Nestler, Dietrich R T Zahn

**Affiliations:** 1 Semiconductor Physics, Technische Universität Chemnitz, D-09107 Chemnitz, Germany; 2 Faculty of Physics, Babes-Bolyai University, Kogalniceanu 1, 400084 Cluj-Napoca, Romania; 3 MEDFUTURE - Research Center for Advanced Medicine, Iuliu Hatieganu University of Medicine and Pharmacy, Louis Pasteur 4–6, 400349 Cluj-Napoca, Romania; 4 Fraunhofer Institute for Electronic Nano Systems, Technologie-Campus 3, 09126 Chemnitz, Germany

**Keywords:** gold nanoparticles, malachite green, microfluidic setup, SERS, silver nanoparticles

## Abstract

A microfluidic setup which enables on-line monitoring of residues of malachite green (MG) using surface-enhanced Raman scattering (SERS) is reported. The SERS active substrate was prepared via laser induced synthesis of silver or gold nanoparticles spot on the bottom of a 200 μm inner dimension glass capillary, by focusing the laser beam during a continuous flow of a mixture of silver nitrate or gold chloride and sodium citrate. The described microfluidic setup enables within a few minutes the monitoring of several processes: the synthesis of the SERS active spot, MG adsorption to the metal surface, detection of the analyte when saturation of the SERS signal is reached, and finally, the desorption of MG from the spot. Moreover, after MG complete desorption, the regeneration of the SERS active spot was achieved. The detection of MG was possible down to 10^−7^ M concentration with a good reproducibility when using silver or gold spots as SERS substrate.

## Introduction

Over the past decade special attention has been given to the investigation of hazardous environmental chemicals with impact on human health [1–4]. Surface-enhanced Raman scattering (SERS) has been proven to be a suitable tool for rapid detection of pollutants [3,5–8], based on their specific molecular fingerprint spectra [9]. The high sensitivity of this spectroscopic technique originates from the pronounced enhancement of the inelastic scattering when the molecule is attached to a rough metallic surface of silver, gold, or copper [10]. While metallic substrates are available in different shapes and sizes [9], colloidal citrate-reduced silver nanoparticles, synthesized following the method by Lee and Meisel [11] under boiling conditions, are amongst the most common employed SERS substrates.

However, the citrate reduced colloids are difficult to be prepared in experiments when in situ synthesis is required. In the past years, miniaturization of the systems became important for the development of bioanalytical sensors, having advantages such as precise control over the processes within the enclosed channels and using small amounts of solutions. The integration of the SERS technique with microfluidic systems [12–13] shows great potential for analytical studies as lab-on-chip SERS. Even if various studies demonstrated the SERS detection within microfluidic devices [12–20], so far, for a successful experiment the procedures require the synthesis of the colloids and previous mixing of the analyte and the silver nanoparticles.

This study focuses on the on-line SERS monitoring of malachite green (MG) as a model analyte. However, the detection at the trace amount level of the selected model analyte MG, is also of interest, because it is used illegally in aquaculture industry to control ectoparasites and fungal infections on fish eggs, fingerlings, and adult fish [21]. In spite of its prohibition, MG is used due to low cost and high efficacy and residues of MG in farmed fish from several countries were reported [22–23].

## Results and Discussion

**Figure 1:**
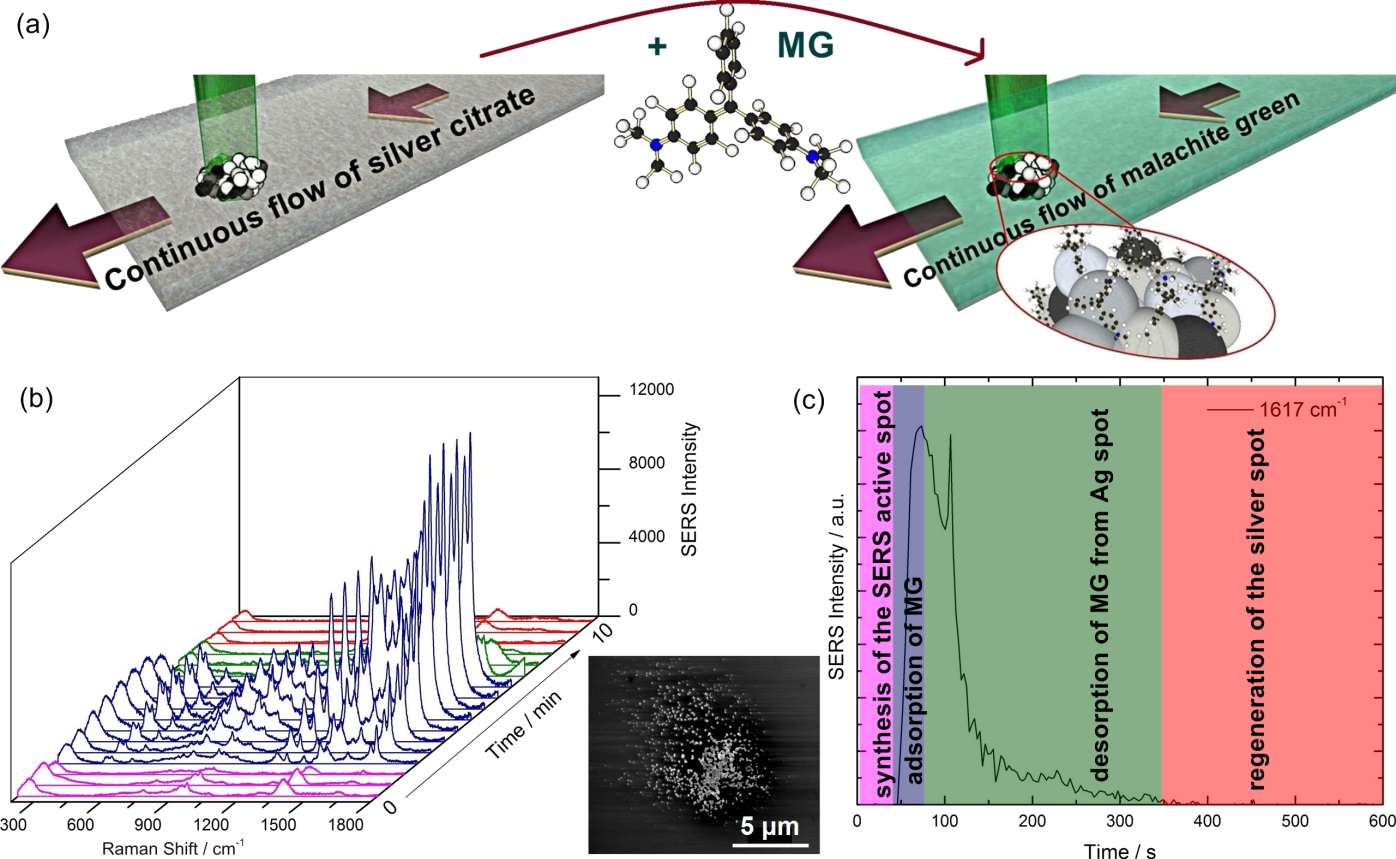
(a) Schematic diagram of the SERS active spot synthesis, followed by MG adsorption to the nanoparticles and SERS detection; (b) the on-line SERS monitoring showing selected spectra recorded during silver spot synthesis, MG adsorption, and desorption using a 10^−5^ M MG solution. The inset from the figure shows a SEM image of the silver spot; (c) the intensity variation of the 1617 cm^−1^ SERS band of MG during the entire process. For all spectra in the figure, the acquisition parameters were 2 accumulations at 1 s exposure.

The SERS detection approach consisted in two sequential processes, both performed in situ: the synthesis of the SERS active spot, formed by silver or gold nanoparticles, followed by SERS detection of the analyte, as shown schematically in Figure 1a.

### SERS monitoring of MG adsorbed on silver nanoparticle spots

The synthesis of the SERS active silver nanoparticles spot was carried out by laser-induced spot synthesis [24] inside the glass capillary. The silver nitrate and sodium citrate solution was injected in a continuous flow into the microfluidic capillary while the laser beam (λ = 514.7 nm) was focused on the bottom part of the capillary. As illustrated in the schematic diagram in Figure 1a, the exposure of the silver/citrate mixture to the laser beam causes the photoinduced synthesis of nanoparticles, building a compact spot on the glass surface [25].

Thus, by focusing the Raman laser beam on the inner walls of the glass capillary filled with a silver/citrate mixture, a spot with a diameter of approximately 7 μm was obtained after 40 seconds exposure. For longer exposure time, no significant changes can be noticed, the size of the spot being determined by the focus area of the used objective [25]. A SEM image of the silver spot synthesized on a silicon surface is shown in the inset of Figure 1b. The spot shows a rough surface, at nanometer scale, which facilitates the SERS effect and detection of analytes.

The successful formation of the SERS active substrate in the microfluidic capillary can be noticed when two of the specific bands of citrate at 953 and 1383 cm^−1^ appear (pink spectra, in Figure 1b), due to the adsorption of citrate ions to the silver nanoparticle surface [24]. The used concentration of citrate was too low in order to be detected by normal Raman spectroscopy prior to the formation of the SERS active spot.

Once the SERS active Ag spot was synthesized inside the glass capillary, the silver/citrate mixture solution flow is stopped and the MG analyte solution, at a concentration of 10^−5^ M, is injected. Due to the high affinity of the investigated molecule for the silver substrate [26], the citrate anions were replaced when MG reached the detection area, leading to the recording of intense SERS spectra (blue spectra, in Figure 1b).

The adsorption of the MG molecule was monitored by recording sequential SERS spectra. The molecules are adsorbed on the surface via a single dimethylamino group in a tilted configuration [27] and, in particular, the specific Raman vibrational bands at 424, 1178, 1366, and 1617 cm^−1^, which are attributed to the out-of-plane modes of phenyl-C-phenyl, in-plane modes of ring CH bending, *N*-phenyl stretching, and ring CC stretching, respectively [28], can be observed in Figure 1b in the blue spectra.

In order to remove MG from the silver surface and to regenerate the SERS substrate, silver/citrate solution was injected again. The desorption of MG from the silver surface is noticed by the decrease of the SERS intensities of MG with time (green spectra, Figure 1b). The full regeneration of the spot is indicated by the reappearance of the citrate bands in the red spectra of Figure 1b.

The adsorption and desorption of MG was monitored during the experiment by watching the SERS intensity at 1617 cm^−1^, corresponding to the highest SERS band of MG, as shown in Figure 1c. The 1617 cm^−1^ band appears after the synthesis of the silver spot (the first 40 seconds in Figure 1c), when MG is injected in the capillary, and adsorbed rapidly to the SERS active substrate. A high increase in the intensity of this band is observed, and after 40 seconds after MG injection, saturation in the recorded SERS intensities is reached, for a flow speed of 5 μL/s.

Desorption of MG from the spot occurs slower than the adsorption, as can be observed in the green part of Figure 1c. The SERS active silver spot is considered regenerated when the citrate SERS bands are observed again (red part in Figure 1c).

**Figure 2:**
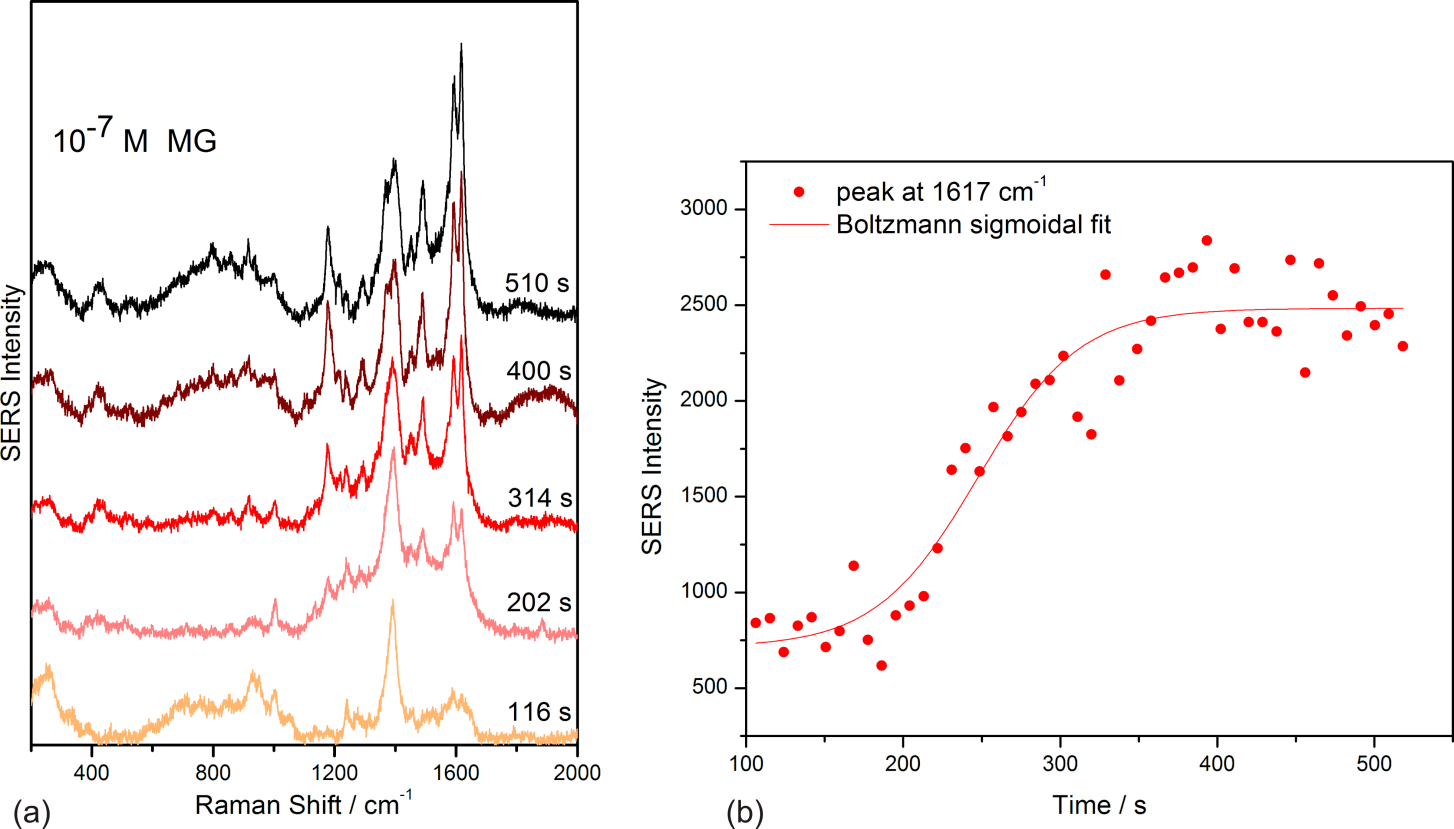
(a) Selected SERS spectra in the time range from 100 to 510 seconds, considered from the start of the experiment (silver/citrate mixture injection), during continuous flow of 10^−7^ M MG in the capillary. The exposure time for recording each spectrum was 4 seconds; (b) time dependence of the SERS intensity of the 1617 cm^−1^ MG marker band.

The SERS detection limit for MG using the microfluidic setup presented here was found to be below 10^−7^ M. When monitoring the SERS spectra of MG at such concentration, the SERS intensity of the marker bands of MG (1178 cm^−1^ and 1617 cm^−1^) increased with the injection time and it reached saturation after approximately 300 seconds of continuous flow of 10^−7^ M MG. Figure 2a shows some of the sequential SERS spectra recorded during injection of the 10^−7^ M MG solution in the capillary.

In the first 100 seconds the silver spot was synthesized, MG being injected after this time range. The time values indicated for each spectrum in Figure 2a are considered from the beginning of the experiment, when silver/citrate mixture is injected in the capillary for the synthesis of the silver spot. After 400 seconds, a saturation of the MG SERS signal is observed, the recorded intensities of the marker bands showing fluctuations, which are typical for SERS experiments, during the continuous flow of 10^−7^ M MG.

The time dependence of the 1617 cm^−1^ band is shown in Figure 2b. The curve, obtained by a Boltzmann sigmoidal fit of the experimental points, can be explained by a Langmuir–Blodgett isotherm [29], describing the adsorption of the analyte in a single layer during the first 100 seconds and in a multilayer during the last 200 seconds while the solution continues flowing.

**Figure 3:**
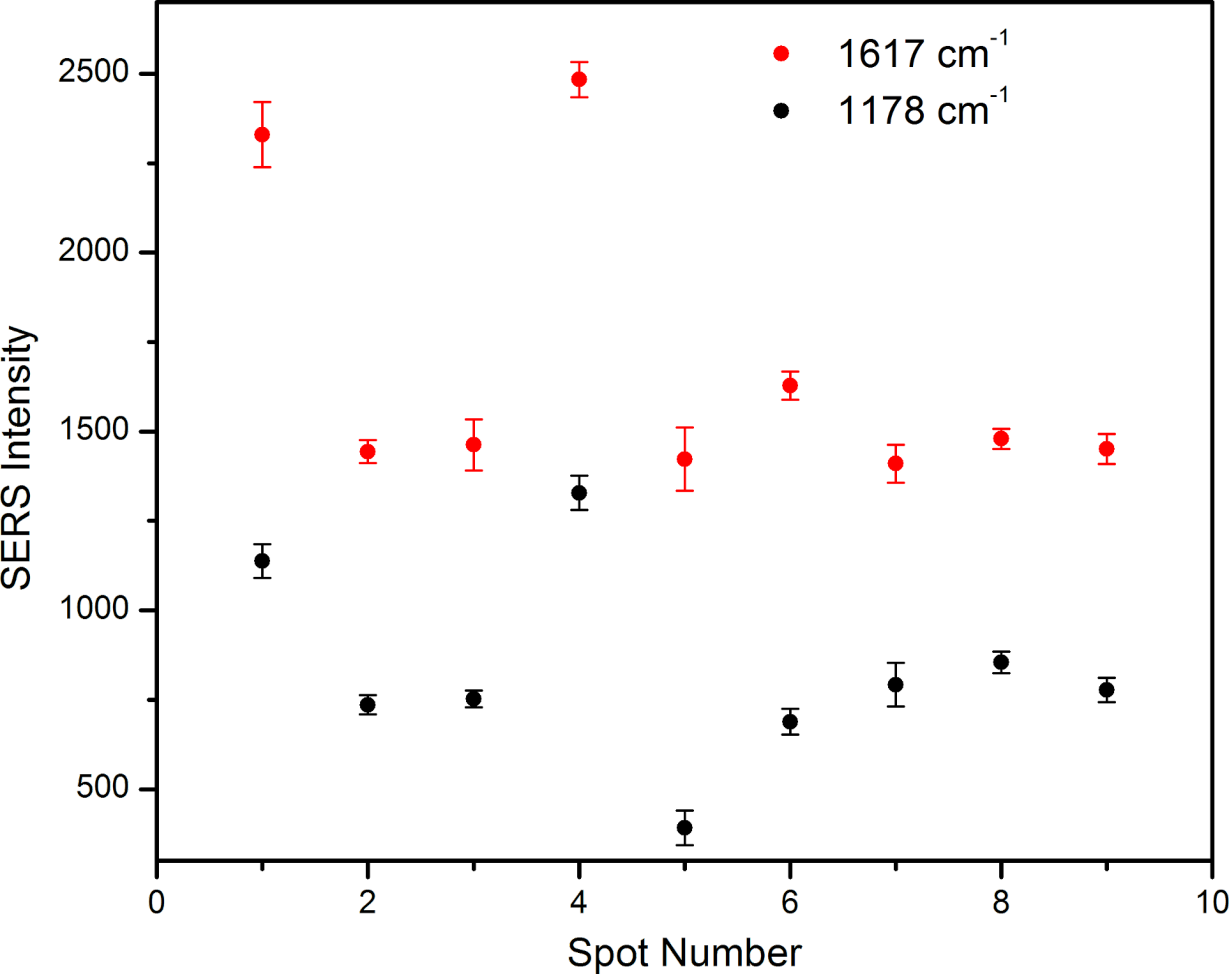
Maximum intensity of the 1178 and 1617 cm^−1^ MG marker bands recorded on nine different silver spots.

In order to test the reproducibility of the MG SERS detection at low concentrations using the proposed microfluidic setup, measurements were performed on freshly prepared spots in new positions, by moving the capillary several μm downstream. Each spot was synthesized in less than 100 seconds. Figure 3 shows the saturation intensity of the MG marker bands at 1178 and 1617 cm^−1^ recorded on nine different spots with a 10^−7^ M MG solution.

A significant deviation from the mean intensity can be observed for the SERS intensity values recorded on spot 1 and 4, however, such deviations are often observed in SERS spectroscopy, as the intensity is very sensitive to the laser focusing on the sample. Still the results show a good reproducibility when comparing the SERS spectra recorded for each spot (Figure 3) and a relative standard error (RSE) of the mean of the intensities of 9% is calculated [30].

### SERS monitoring of MG adsorbed on gold nanoparticle spots

The adsorption and desorption process of MG was studied as well on a SERS active gold substrate. Even though the Raman signal enhancement of gold is lower than silver, this material is often employed in SERS bioanalytical studies [31]. The SERS detection limit for MG using the microfluidic setup with gold substrate was found to be 10^−7^ M.

**Figure 4:**
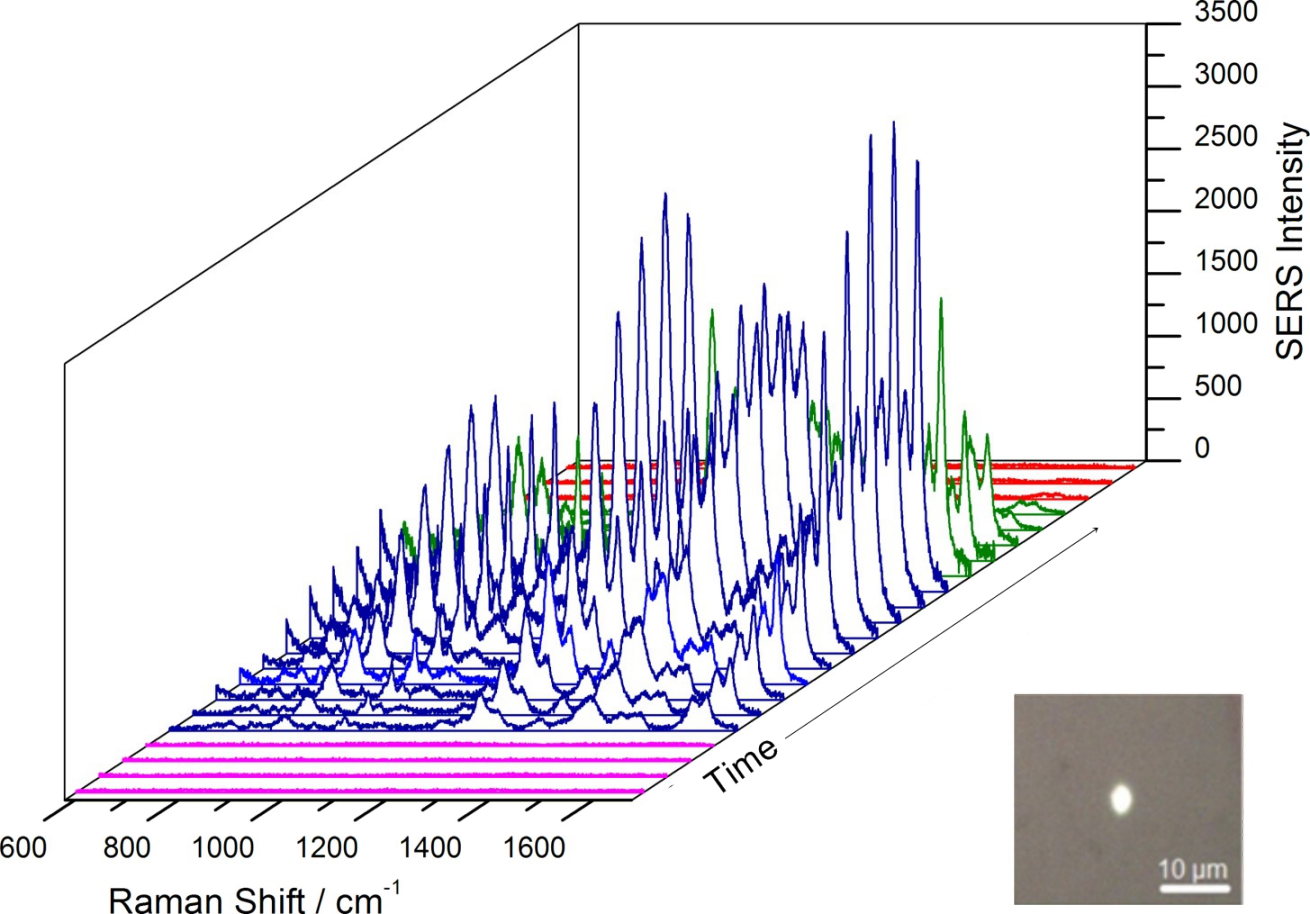
On-line SERS monitoring showing selected spectra recorded during the in situ synthesis of the gold spot, the adsorption, and desorption of 10^−5^ M MG solution on the substrate. The inset from the figure shows an optical image of the gold spot on the glass capillary surface.

The synthesis of the gold spot followed the same procedure as for the silver spot: the laser beam (λ = 632.8 nm) was focused on the continuous flow of gold/citrate mixture injected for several seconds leading to the synthesis of the gold spot on the bottom part of the capillary. Afterwards, in order to detect and monitor the adsorption and desorption of the analyte on the gold substrate, MG was injected in the capillary. Figure 4 shows selected sequential SERS spectra recorded during the synthesis of the gold spot, adsorption and desorption of MG from the SERS active surface.

While during the silver spot synthesis the appearance of specific bands of citrate in the spectra (Figure 1) indicated the successful preparation of the SERS active substrate, in the case of gold no significant Raman signal of citrate could be recorded (Figure 4). Parameters such as exposure time (10 to 300 seconds) and injection flow (1 to 10 μL/s) were investigated in order to determine the optimum parameters for the synthesis of the gold substrate. Optical images recorded after each experiment suggest that 60 seconds of laser exposure on a continuous flow of 1 μL/s leads to the successful preparation of the SERS active gold substrate (inset in Figure 4). Afterwards the 10^−5^ M MG solution was injected in the system. Sequential SERS spectra show the adsorption of the analyte to the gold substrate within 50 seconds. Desorption of the molecule from the gold substrate is noticed within 200 seconds when gold/citrate solution is injected in the capillary (Figure 4, green spectra). The regeneration of the substrate was noticed when the gold/citrate mixture was injected for another 300 seconds and no SERS signal of MG was observed anymore.

## Conclusion

We reported a straightforward approach for on-line preparation of silver and gold nanoparticle spots as SERS active substrates, which was used for fast detection of the model compound MG. By using silver or gold spots as SERS substrate, the detection of MG was possible down to 10^−7^ M concentration, with a good reproducibility, a RSE of 9% being calculated for the SERS saturation intensity, by performing measurements on nine silver spots. The described microfluidic setup enables within a few minutes the monitoring of several processes: the synthesis of the SERS active spot, MG adsorption to the metal surface, detection of the analyte, and finally the desorption of MG from the spot. Moreover, after MG complete desorption, the regeneration of the SERS active spot was achieved.

A main advantage of the she silver and gold nanoparticle spots is the simple and rapid preparation methodology for obtaining these SERS substrates in situ. The silver spot showed a higher Raman enhancement compared to the gold spot, when using malachite green as test molecule.

The results obtained are promising for the development of lab-on-chip approaches for the detection and on-line monitoring of pollutants in water, based on this straightforward and effective microfluidic SERS setup.

## Experimental

**Figure 5:**
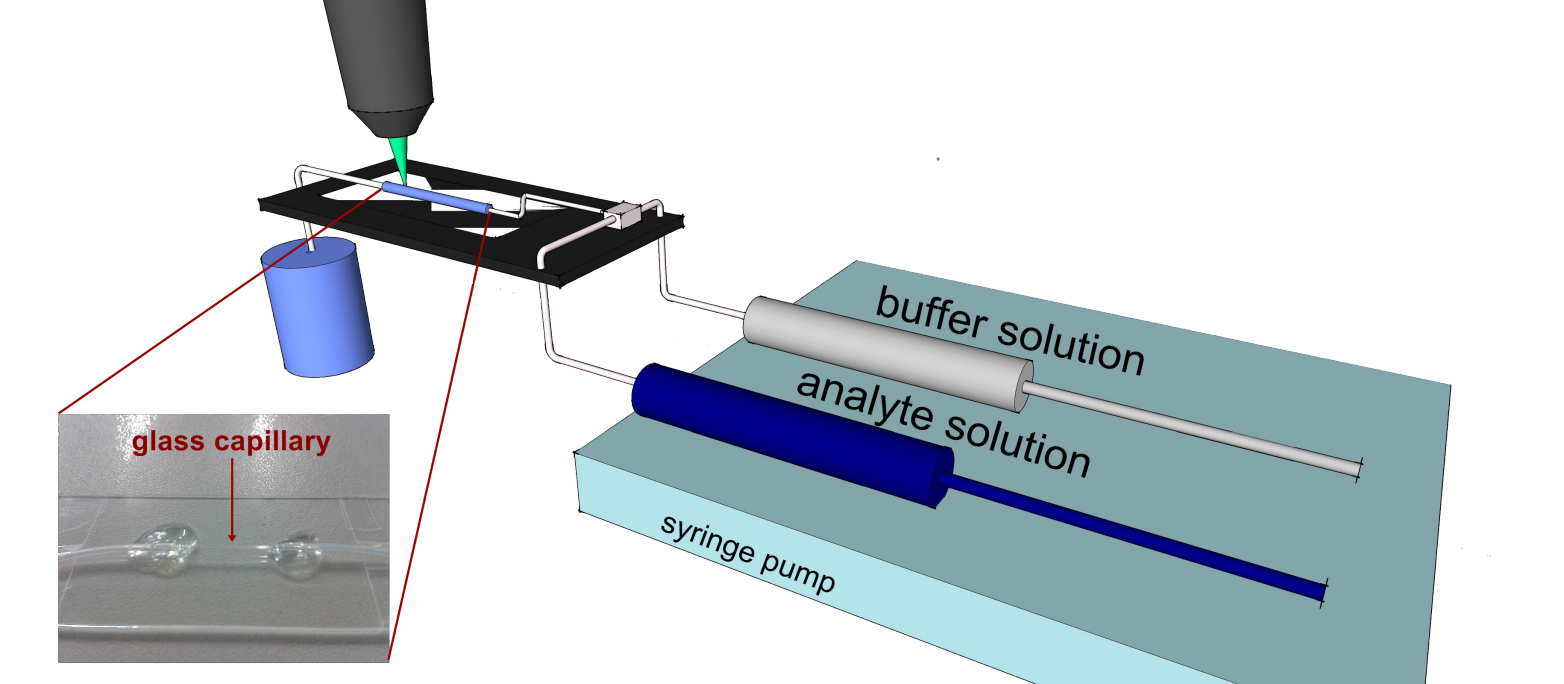
Schematic representation of the microfluidic SERS setup. The inset shows a picture of the glass capillary.

A schematic representation of the SERS microfluidic setup is shown in Figure 5. The experiments were carried out in a square glass capillary with 200 μm inner dimension, 400 μm outer dimension, and 100 mm length (WaleApparatus) as shown in the inset of Figure 1. The capillary was connected with two 10 mL plastic syringes (NeoLab) through PTFE tubes (0.56 mm × 1.07 mm/0.81 mm × 1.63 mm). The two syringes filled with the silver or gold/citrate mixture and MG solution, respectively, were controlled by a NEMESYS low pressure syringe pump (Cetoni GmbH) using NEMESYS UserInterface (Cetoni GmbH). The flows were set to be 3.3 μL/s in the case of the silver/citrate mixture, 1 μL/s for the gold/citrate mixture, and 5 μL/s for the MG solution.

The Raman measurements were performed using a micro confocal Raman system (LabRam HR800 Horiba JY), equipped with a solid state Cobolt laser source operating at 514.7 nm and a 632.8 nm HeNe laser source. The SERS spectra were recorded using a diffraction grating of 600 lines/mm and a nitrogen cooled CCD with a spectral resolution of 2.3 cm^−1^. The laser power measured under a 50× long working distance objective with numerical aperture of 0.5 was 0.30 mW for the 514.7 nm laser source and 3 mW for the 632.8 nm laser source.

For characterizing the SERS active substrate, a silver spot was prepared on a silicon surface by focusing the laser beam (λ = 514.7 nm) on 5 μL of silver/citrate mixture for 60 s. The scanning electron microscopy (SEM) image was obtained with a FEI NovaNanoSEM 200 scanning electron microscope with a working distance of 5.9 mm.

The following chemical reagents were used: silver nitrate (Alfa Aesar), hydrogen tetrachloroaurate(III) hydrate 99.9% (Alfa Aesar), sodium citrate tribasic dihydrate (Fluka Analytical) and malachite green oxalate (Merck). The silver/citrate solution was prepared as previously described [32] by mixing 1 mM silver nitrate and 10 mM sodium citrate, both chemicals being first solved in ultrapure water with a resistivity of 18 MΩ∙cm. The gold/citrate solution was prepared by solving 60 μL of 2% gold salt solution (1 g in 50 mL water) and 300 μL of 1% sodium citrate solution (1 g in 100 mL water) in 9.7 mL of ultrapure water. The silver/citrate mixture was kept in a dark glass bottle in order to avoid silver colloid formation when exposed to light, while the gold/citrate mixture was freshly prepared before each experiment. The investigated analyte was prepared by dilution of MG powder in ultrapure water until the desired concentration was obtained. All MG solutions were freshly prepared before the measurements.
